# A207 CLONAL PATTERNS BETWEEN POUCH NEOPLASIA AND PRIOR COLORECTAL NEOPLASIA IN INFLAMMATORY BOWEL DISEASE PATIENTS: AN EXPLORATORY COHORT STUDY

**DOI:** 10.1093/jcag/gwac036.207

**Published:** 2023-03-07

**Authors:** L Van Lierop, M te Groen, L Derikx, B Ylstra, F Hoentjen, I Nagtegaal, F Doubrava-Simmer

**Affiliations:** 1 Gastroenterology, University of Alberta, Edmonton, Canada; 2 Gastroenterology, Radboud University Medical Center, Nijmegen; 3 Gastroenterology, Erasmus Medical Center, Rotterdam; 4 Medicine, Amsterdam UMC, Amsterdam; 5 Pathology, Radboud University Medical Center, Nijmegen, Netherlands

## Abstract

**Background:**

Inflammatory bowel disease (IBD) patients with an ileo-anal pouch anastomosis (IPAA) bear an increased risk of pouch neoplasia, with prior colorectal neoplasia as the strongest predictor. It is unknown if pouch neoplasia develops independently or is derived from prior colorectal neoplasia.

**Purpose:**

We aimed to assess potential clonality between prior colorectal neoplasia and pouch neoplasia in IPAA patients with IBD.

**Method:**

In this explorative study we used the Dutch Nationwide Pathology Databank to identify IBD patients with both pouch neoplasia and colorectal neoplasia prior to colectomy. Clonality was assessed on colonic tissue of the lesion with shallow whole genome sequencing based copy number aberration (CNA) analysis and validated with immunohistochemistry (IHC) and fluorescence in situ hybridization (FISH).

**Result(s):**

We included 13 patients who fulfilled the inclusion criteria. Three patients showed matching clonality CNA profiles between prior colorectal neoplasia and pouch neoplasia, validated with matching IHC and FISH for p19 and HER2. Patients with matching clonal samples also showed on retrospective review concordant histology of the neoplastic lesion pre- and post IPAA, positive resection margins, metastasized disease or a short interval (<2 years) between colorectal and pouch neoplasia diagnoses.

**Image:**

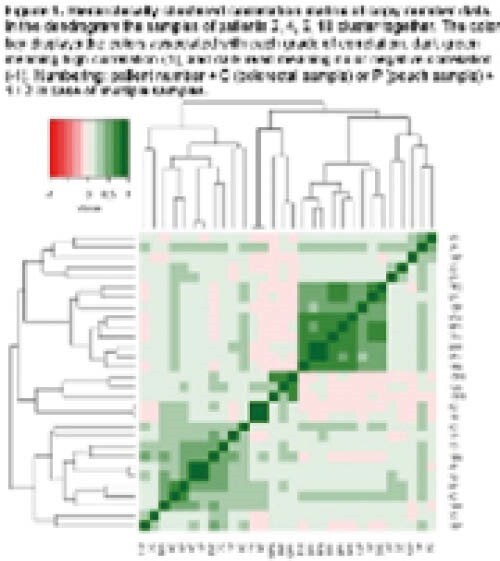

**Conclusion(s):**

Three patients showed matching clonality patterns of neoplastic lesions, confirmed by clinical and histological data. Most pouch neoplasia in our cohort were molecularly different from their prior colorectal neoplasia. CNA provides a feasible method for clonality assessment in patients with colorectal neoplasia and subsequent pouch neoplasia.

**Disclosure of Interest:**

None Declared

